# The role of processing efficiency and selection history in the limit of visual awareness in shape perception

**DOI:** 10.1167/jov.22.8.9

**Published:** 2022-07-15

**Authors:** Makayla Szu-Yu Chen, Caitlin Megan Roscherr, Zhe Chen

**Affiliations:** 1 School of Psychology, Speech, and Hearing, University of Canterbury, Christchurch, New Zealand; 2 School of Psychology, Speech, and Hearing, University of Canterbury, Christchurch, New Zealand; 3 School of Psychology, Speech, and Hearing, University of Canterbury, Christchurch, New Zealand

**Keywords:** processing efficiency, selection history, attentional control setting, visual attention, unit of access

## Abstract

A growing body of research indicates that the limit in instant conscious awareness, or the unit of access, for some object features such as color, orientation, and direction of motion is more than one. In four experiments we explored the roles of processing efficiency and selection history in shape perception. Two targets, which were geometric shapes (less efficient) or alphabet letters (more efficient), were shown simultaneously or sequentially. The task was to judge whether a test probe matched one of the targets. In different experiments, the two types of trials were presented in separate blocks, interleaved couplets, or randomly within the same block during testing but regrouped in data analyses such that the same type of trials was either repeated or not repeated. Accuracy was higher in the sequential than simultaneous trials for geometric shapes, but not for upright letters, when the same trial type was blocked or in the repeated condition. These results suggest that processing efficiency and selection history both play a role in the unit of access in shape perception. They also underscore the flexibility of the visual system, which uses different factors to maximize conscious visual perception at a given time.

## Introduction

How many different colors or shapes we can consciously perceive at a given moment is a topic of much debate. Some researchers have proposed that, although we can have instant conscious awareness of, or access to, multiple locations, the unit of access for object features such as color, shape, size, and orientation is limited to one feature value per dimension (Huang & Pashler, 2007; Huang, Treisman, & Pashler, 2007). In other words, if a display consists of a blue square and a red triangle, then we can consciously perceive two locations, but only one of two colors (blue or red) or one of two shapes (square or triangle) at a given instant.

This asymmetry was demonstrated by Huang and colleagues in a series of experiments using a simultaneous/sequential paradigm (e.g., Huang & Pashler, 2007; Huang et al., 2007). Participants saw two color squares followed by a test probe. The squares were briefly presented either simultaneously or sequentially in alternate displays. The task was to judge whether the probe matched the color or location of one of the targets. In the location task, performance was comparable between the two types of trials, indicating simultaneous access to two locations. However, in the color task, accuracy was lower in the simultaneous trials than in the sequential trials, suggesting that only one color was perceived at a time. Consistent with the latter result, Becker and colleagues (Becker, Miller, & Liu, 2013; Miller, Becker, & Liu, 2014) found poorer performance when two targets were presented simultaneously rather than sequentially in an orientation task, indicating single-feature access in orientation perception.

However, there have also been studies that reported different results (e.g., Chen & Chen, 2021; Fitousi, 2019; Hao, Becker, Ye, Liu, & Liu, 2018; Mance, Becker, & Liu, 2012; Miller et al*.*, 2014; Rideaux, Apthorp, & Edwards, 2015). Mance et al. (2012) noted several built-in contingencies in the original simultaneous/sequential paradigm. When these contingencies were removed, the difference in performance for color between the two types of trials disappeared, indicating that two colors were accessed at the same time. Miller et al*.* (2014) also found parallel access for color but single access for orientation. Using a different paradigm, Hao et al. (2018) reached a similar conclusion. They measured participants’ event-related potentials while the participants memorized a briefly presented display that consisted of one, two, or four different color patches or gratings of different orientations in each hemifield. Interestingly, the amplitude of the contralateral delay activity, which is an electrophysiological marker for the number of items that can be consolidated (Woodman & Vogel, 2008), increased from one stimulus to two stimuli per display when the task was color but remained the same when the task was orientation. These results indicate that, compared with orientation, more colors could be consolidated at a time.

Chen and Chen (2021) further demonstrated that the unit of access in color perception was modulated by participants’ prior experience within a task. Accuracy was higher in the sequential trials than in the simultaneous ones when the two types of trials were presented in interleaved couplets but not in separate blocks. Moreover, when all the trials were mixed randomly within a block during testing but were regrouped in data analyses such that the same type of trials was either repeated (repeat condition) or not repeated (switch condition), performance was comparable between the simultaneous and sequential trials in the repeat condition. These results showed that a recent history of attentional deployment can induce selection biases that may not be optimal for the task at hand. These effects of “selection history” raised the question about the generality of single-feature access in color perception.

Selection history is known to influence the attentional control setting in a variety of tasks (Leber & Egeth, 2006; Leber, Kawahara, & Gabari, 2009; Maljkovic & Nakayama, 1994; Talcott & Gaspelin, 2020; Yeh, Lee, Chen, & Chen, 2014). Factors such as response strategies, the size of attentional zoom, and the allocation of attentional resources can all be affected by participants’ prior experience (Chen, 2003; Chen & Cave, 2013; Chen & Cave, 2016; for a review, see Awh, Belopolsky, & Theeuwes, 2012). When the same type of trials was presented in succession, such as in the block and repeat conditions in Chen and Chen (2021), an optional attentional setting could be used from trial to trial, resulting in comparable performance between the simultaneous and sequential trials. However, when the type of trials changed from trial to trial, it became too costly or impossible to deploy an optimal attentional setting on a given trial. As the simultaneous trials required more attentional resources or greater effort, performance was impaired in the simultaneous condition relative to the sequential one.

Consistent with the above account, there is evidence that encouraging spatial attention (i.e., attention that prioritizes one region of space over other regions), can increase the number of orientations reaching awareness. Rideaux et al. (2015, Experiment 3) found that, when the locations of the targets changed from trial to trial, accuracy was higher in the sequential condition than in the simultaneous condition. However, when the locations of the targets were fixed within a block, no difference in performance was observed between the two types of trials. These results indicate that prior knowledge about the locations of the targets can facilitate the deployment of spatial attention to the targets, and this in return can increase the unit of access in orientation perception.

In addition to spatial attention, similarity among the targets has also been found to affect the unit of access. Rideaux et al. (2015, Experiments 1 and 2) showed in two motion direction matching tasks that the number of directions that could be perceived simultaneously was influenced by the range of the motion directions. When the four possible directions occupied a large range such that there was little confusion about the direction of motion for each target, no difference in performance was found between the simultaneous and sequential trials. However, when the range was reduced so that the motion direction became more difficult to discriminate, accuracy was higher in the sequential trials than in the simultaneous ones.

Both similarity among the targets and knowledge about the target locations are associated with how efficiently (typically defined as how fast and/or how accurately) the targets can be processed (but see below for the operational definition of processing efficiency in the present study). That the processing efficiency of the targets should influence the unit of access makes sense. In a typical simultaneous/sequential paradigm, stimulus displays are presented very briefly. For targets to have durable representations so that they can be accessed consciously, encoding must be fast. If one set of stimuli requires less time during the encoding stage or during the target–probe comparison stage than another set of stimuli, more stimuli in the former set can be encoded within a given time, resulting in an increase in the unit of access. Consistent with the above reasoning was the finding that participants were less accurate in change-detection tasks when stimulus complexity increased (Alvarez & Cavanagh, 2004) or when the similarity between the target and test arrays increased (Awh, Barton, & Vogel, 2007). Similarly, the time to find a target was also longer in visual search tasks when there was high similarity between the target and distractors (Duncan & Humphreys, 1989).

In the present study, we investigated the roles of processing efficiency and selection history in shape perception. These factors had been examined separately in the perception of motion direction and color, respectively. However, to our knowledge, no systematic research had been conducted to determine whether the two factors would jointly influence the unit of access. We chose a shape task partly because little was known about the unit of access for shape perception and partly because shape is arguably the most important feature in object identification. Although most objects can be recognized by shape alone, the same cannot be said for color, orientation, or motion.

In the experiments reported here, processing efficiency was operationally defined as the target exposure duration required to reach a preset criterion based on accuracy (see details below), with shorter duration indicating more efficient processing. As in previous research, we used a simultaneous/sequential paradigm. Across different experiments, we manipulated processing efficiency by requiring participants to respond to geometric shapes (less efficient) or alphabet letters (more efficient). Following Chen and Chen (2021), we examined selection history by showing the simultaneous and sequential trials in separate block, interleaved couplets, or by mixing the two types of trials within the same block during testing, but re-grouping them into repeat and switch conditions in data analyses. Of specific interest was whether participants would perceive more alphabet letters than geometric shapes at a given time, and whether this pattern of data would depend on participants’ selection history.

## Experiments 1A and 1B

**Figure 1.**
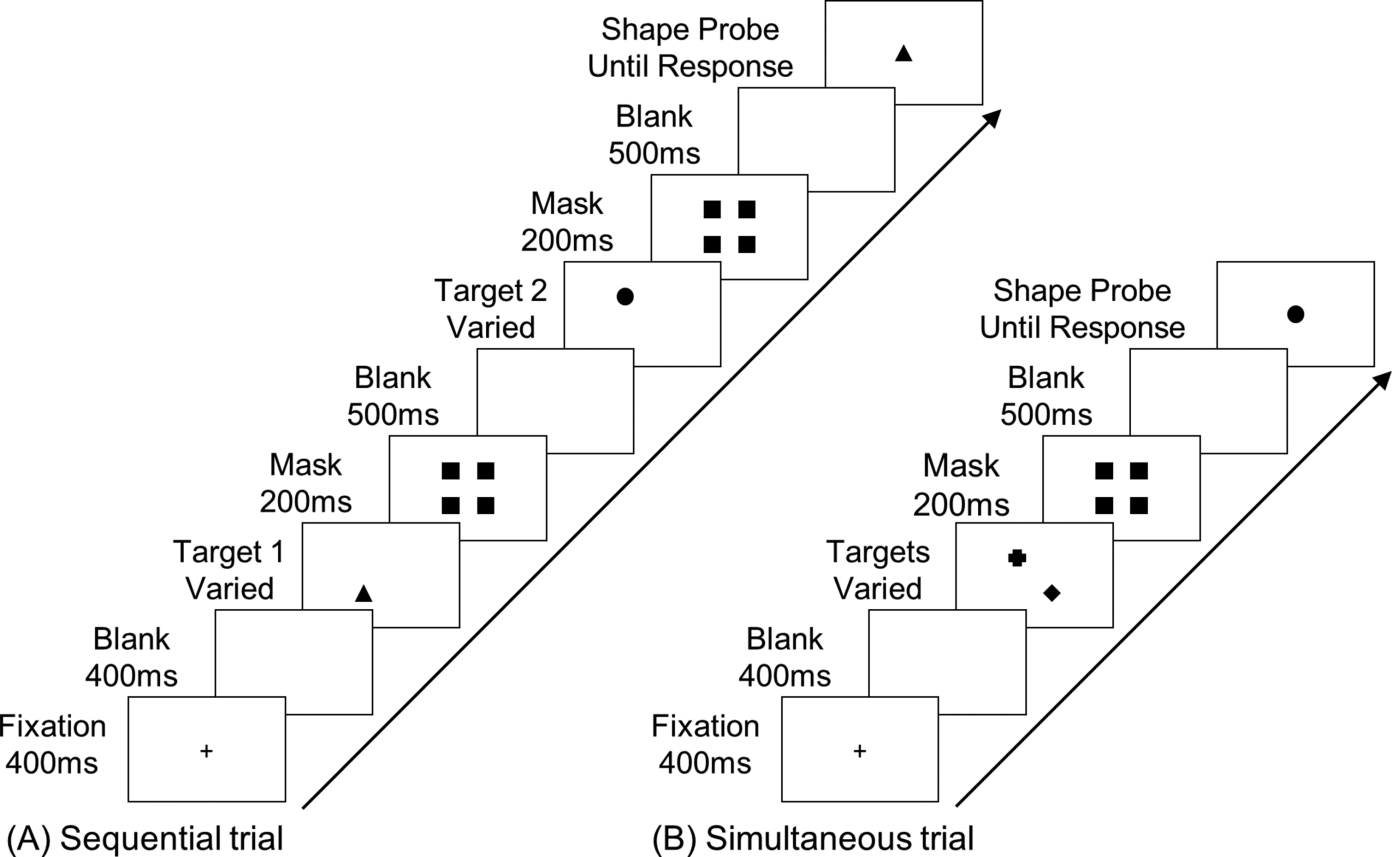
Examples of trials from Experiments 1A and 1B. (A) A sample trial of the sequential condition. (B) A sample trial of the simultaneous condition.

Experiments 1A and 1B (see Figure 1) were modeled after the two experiments in Chen and Chen (2021). Unlike our previous study, in which the target set consisted of four highly discriminable color patches, in the present experiments we used four geometric shapes that were less discriminable. As before, two targets were presented either simultaneously or sequentially. In Experiment 1A, the two types of trials were shown in separate blocks (i.e., block condition) or in interleaved couplets (i.e., interleave condition). In Experiment 1B, they were randomly mixed within a block in testing but were regrouped in data analyses such that the same type of trials was either repeated (i.e., repeat condition) or not repeated (i.e., switch condition). Given that the processing of the geometric shapes was not expected to be very efficient, we predicted higher accuracy in the sequential trials than in the simultaneous ones in the interleave condition in Experiment 1A and in the switch condition in Experiment 1B. What was less clear was the effect of selection history: whether it would eliminate the difference in performance between the two types of trials when the same attentional set could be used from trial to trial in the block condition in Experiment 1A and in the repeat condition in Experiment 1B.

### Experiment 1A

#### Method

##### Participants

Thirty undergraduate students from the University of Canterbury participated in the experiment for course credits. This sample size was based on Chen and Chen (2021, Experiment 1), who reported an effect size of η*_p_*^2^ = 0.17 for the interaction between trial type (simultaneous vs. sequential) and grouping (block vs. interleave). Assuming the same effect size, with α = 0.05 and 80% power, G*Power 3.1 (Faul, Erdfelder, Buchner, & Lang, 2009) recommended a sample size of 26. A sample size of 30 was deemed sufficient.

##### Apparatus and stimuli

All stimuli were shown on a PC with a 24-inch monitor. E-Prime 3.0 was used to present the stimuli and record responses. Participants were tested individually in two dimly lit rooms. The viewing distance was approximately 60 cm. All of the stimuli were black presented against a white background. Each trial started with a fixation cross, followed by one or two target displays depending on the experimental condition, a mask after each target display, and finally a probe. The fixation, which was located at the center of the screen, subtended 0.3° in length and width. The target set consisted of four geometric shapes: a cross, a diamond, a circle, and a triangle. Each shape subtended 1.6° × 1.6° and was equally likely to be presented at one of four possible target locations at the corner of a centrally located, invisible square of 4.8° per side. On simultaneous trials, two shapes were selected randomly without replacement from the target set, and they were shown concurrently in the same display. On sequential trials, the targets appeared one at a time in two separate displays. The mask consisted of four identical black squares, each having the same size as that of the target, and the four squares were presented at the four possible target locations. The probe was one of the stimuli in the target set, and it was always shown at the center.

##### Design and procedure

The experiment used a 2 × 2 repeated-measures design. The two factors were Grouping (block vs. interleave) and Presentation (simultaneous vs. sequential). Each trial began with the fixation for 400 ms, followed by a blank screen of 400 ms, and then the onset of the target display for a varied duration (see details below). In the simultaneous condition, which consisted of 50% of the trials, two targets would appear concurrently, and their offset would be followed by a 200-ms mask display. In the sequential condition, one target would appear at a time, and each would be followed by a mask of 200 ms. On any given trial, the locations of the targets were randomly selected, and each location was equally likely to be chosen as a target location. In both conditions, upon the offset of the final (or the only) mask, the screen would be blank for 500 ms before the onset of the test probe, which would remain on the screen until response. On half of the trials, the probe matched one of the targets. On the rest of the trials, it was equally likely to be one of the remaining stimuli in the target set. Participants were instructed to press the “J” key if the probe matched one of the targets, and to press the “K” key otherwise. Accuracy, but not speed, was emphasized.

Accuracy was assessed every 12 trials. Regardless of the experimental condition, the initial duration was set at 116 ms. If the average accuracy from the previous 12 trials was higher than 75%, there was a decrease of 17 ms. If accuracy was lower than 65%, the duration increased by 17 ms. The minimum duration was 33 ms and the maximum was 200 ms.

Each participant completed two sessions of trials at one sitting. In one session (i.e., block condition), there were three simultaneous and three sequential blocks, with each block consisting of 48 trials. The order of the blocks was randomized across participants. However, participants were informed of the type of trials before each block. In the other session (i.e., interleave condition), the two types of trials were presented in couplets, and the first of the couplet was always a sequential trial. The order of the session was counterbalanced across participants. Altogether, there were 576 trials divided equally between the block condition and the interleave condition. Participants completed 12 practice trials before each session.

#### Results and discussion

Trials with reaction times less than 200 ms or greater than 20 seconds were excluded from analysis, and the same data treatment was used in all the experiments in this study. The data of two participants were excluded, one due to high anticipatory responses (over 15% of trials faster than 200 ms in one session), and the other for chance performance (mean error rate of 51.5%).

**Figure 2.**
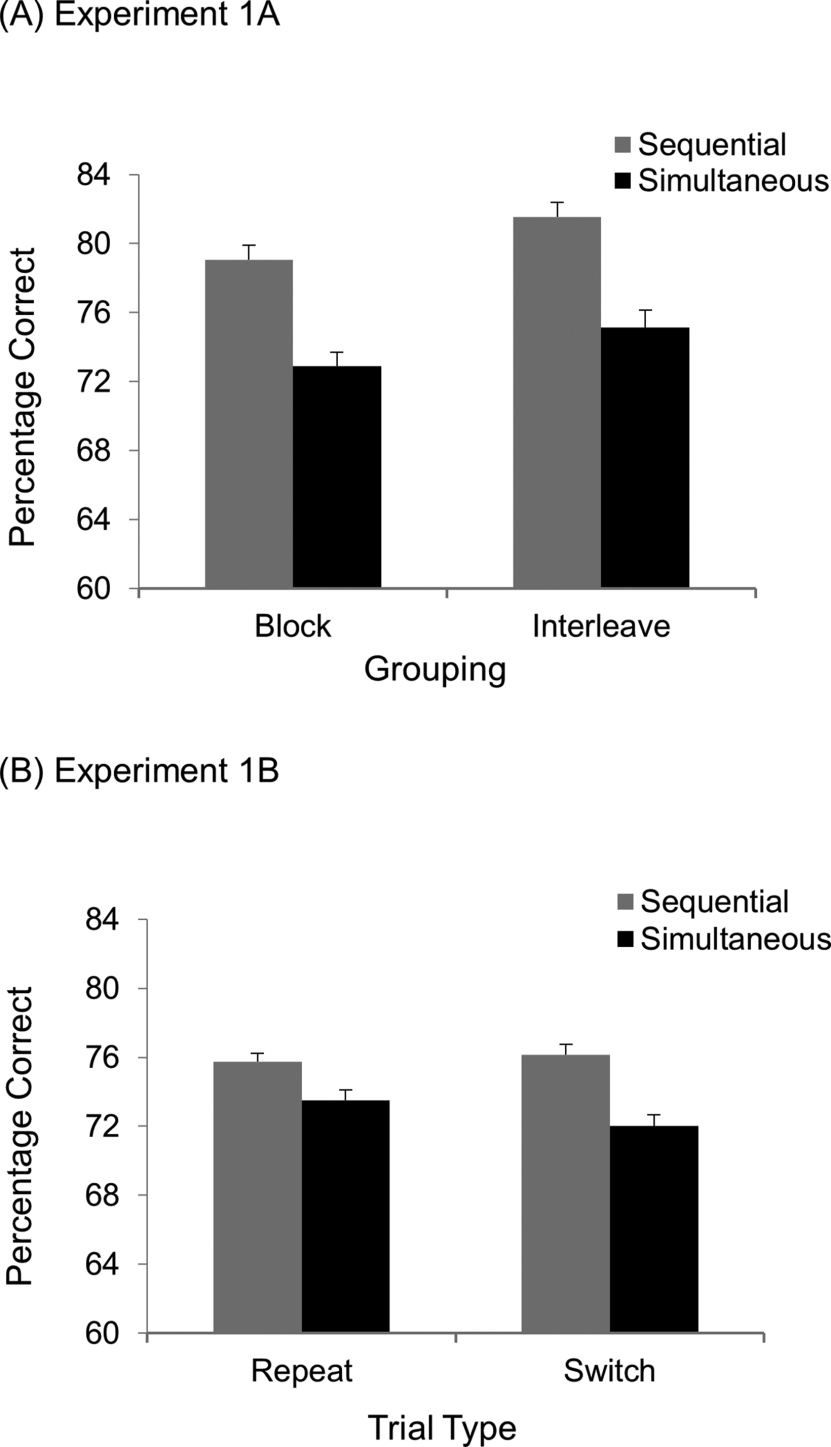
Results from Experiments 1A and 1B. Mean accuracy (percent correct) and SEs as a function of Grouping and Presentation in Experiment 1A (A), and Trial Type and Presentation in Experiment 1B (B).

The mean target exposure duration was 73.8 ms (range, 43.1–188.4; *SD*, 42.4 ms) for the interleave session and 79.6 ms (range, 43.8–177.6; *SD*, 43.5 ms) for the block session. The accuracy data are shown in Figure 2. All of the figures in this article show mean accuracy with an error bar of +1 within-subjects standard error of the mean (Cousineau, 2005). A 2 × 2 repeated-measures analysis of variance (ANOVA) on the accuracy data revealed a significant main effect of Presentation, indicating higher accuracy in the sequential condition (80.3% correct) than in the simultaneous condition (74.0% correct), *F*(1, 27) = 47.47, *MS_e_* = 23, *p* < 0.001, η*_p_*^2^ = 0.64. Neither Grouping nor its interaction with Presentation was significant: *F*(1, 27) = 3.53, *MS_e_* = 44, *p* = 0.071, and η*_p_*^2^ = 0.12 for Grouping; *F*(1, 27) < 1 (not significant) for Grouping by Presentation interaction. These results indicate a unit of access of one for geometric shapes. Furthermore, there is no indication that selection history played a role in performance. We will discuss the results more in Experiment 1B.

### Experiment 1B

#### Method

The method of Experiment 1B was the same as that of Experiment 1A except for the presentation of the target stimuli. Instead of appearing in separate blocks, simultaneous and sequential trials were randomly intermixed within a block and were selected with equal probability on a given trial. As before, the experiment consisted of 576 experimental trials, and they were divided into six equal blocks. Participants were encouraged to take a short break after each block. Thirty new undergraduate students took part in the experiment.

To further examine the role of selection history in the perception of geometric shapes, we regrouped the trials in data analyses into a repeat condition and a switch condition. In the repeat condition, the same type of trials appeared in *n* and *n* – 1 trials (i.e., a simultaneous trial preceded by another simultaneous trial, or a sequential trial by another sequential one). In the switch condition, the trial type differed in *n* and *n* – 1 trials.

#### Results and discussion

The mean target exposure duration was 76.9 ms (range, 39.5–185.0; *SD*, 36.9 ms). A 2 × 2 repeated-measures ANOVA with Trial Type (repeat vs. switch) and Presentation (simultaneous vs. sequential) was performed on the accuracy data. As before, there was a reliable effect of Presentation, with higher accuracy in the sequential condition (75.9% correct) than in the simultaneous condition (72.7% correct), *F*(1, 29) = 17.48, *MS_e_* = 17, *p* < 0.001, η*_p_*^2^ = 0.38. Neither a main effect of Trial Type nor its interaction with Presentation was found: *F*(1, 29) < 1 (not significant) and *F*(1, 29) = 2.29, *MS_e_* = 11, *p* = 0.141, and η*_p_*^2^ = 0.07 for Trial Type and Trial Type by Presentation interaction, respectively. These results provided no evidence that Trial Type influenced task performance.

Once again, performance was superior in the sequential condition than in the simultaneous condition, suggesting that the unit of access was limited to one item for geometric shapes. These results are consistent with the findings in Experiment 1A, which also showed higher accuracy in the sequential trials than in the simultaneous ones.

It is notable that no evidence was found in either experiment that prior knowledge or selection history influenced participants’ performance. Performance was significantly worse in the simultaneous condition than in the sequential condition even when the two conditions were blocked in Experiment 1A. Although participants could in theory use different attentional control settings in the two conditions (e.g., by using different extent of attentional zoom or different level of effort), the absence of a significant difference in performance between the two conditions suggests that either the same attentional control setting was used in both conditions or the effect of different attentional control setting on performance was negligible.

The pattern of data in Experiments 1A and 1B differed markedly from the pattern of data in Chen and Chen (2021), whose participants completed a color task and showed comparable performance between the sequential and simultaneous trials in both the block and repeat conditions. As the colors used in that study were highly discriminable, their processing was likely to be efficient, making it possible for two stimuli to be processed simultaneously when an optimal attentional control setting could be deployed. If processing efficiency indeed played a key role in the different pattern of data between the present experiments and the study by Chen and Chen (2021), then making the target stimuli more discriminable should also increase the limit of access in shape perception. Experiment 2 tested this hypothesis.

## Experiment 2

In Experiment 2, the targets were alphabet letters instead of geometric shapes. As letters are ubiquitously used in everyday life and the ones we selected had a low level of similarity among them, their processing should be quite efficient. Based on previous research, we expected participants to have access to more than one shape at a time, especially in the repeat condition where they could use the same attentional control setting in successive trials.

###  

#### Method

The method was the same as that of Experiment 1B except for the following differences. First, all stimuli were white against a black background.^1^ Second, the target set consisted of four uppercase letters (T, X, S, and G) written in bold, 54-point Consolas font. The mask, which subtended 1.6° × 1.6°, was made of different features of the letters. As in Experiment 1B, the simultaneous and sequential trials were randomly intermixed within a block, and the data were re-grouped into the repeat and switch conditions in data analyses. Forty-four naïve participants took part in the experiment.^2^

#### Results and discussion

**Figure 3.**
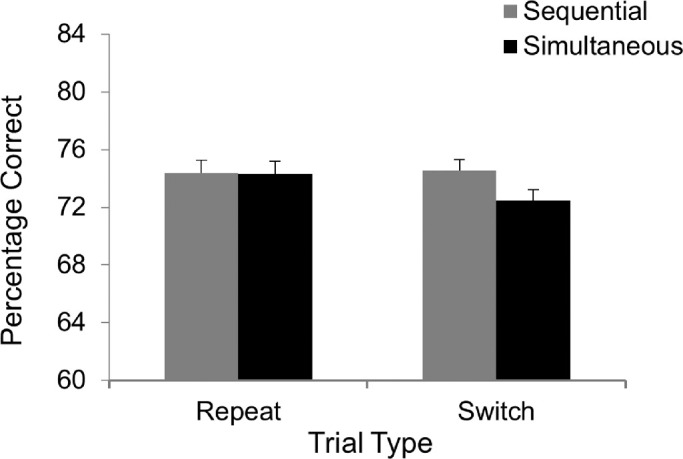
Results from Experiment 2.

The data of two participants were excluded, one due to a high rate of anticipatory responses (>27%) and the other for high error rates (47% averaged across all conditions). The mean target exposure duration was 54.8 ms (range, 38.1–109.1; *SD*, 15.3 ms). Figure 3 shows the accuracy data. A 2 × 2 repeated-measures ANOVA indicated no significant difference in accuracy between the simultaneous and sequential trials, *F*(1, 41) = 1.41, *MS_e_* = 35, *p* = 0.242, η*_p_*^2^ = 0.03, or between the repeat and switch conditions, *F*(1, 41) = 2.04, *MS_e_* = 15, *p* = 0.161, η*_p_*^2^ = 0.05. However, the interaction between Presentation and Trial Type was significant, *F*(1, 41) = 4.42, *MS_e_* = 10, *p* = 0.042, η*_p_*^2^ = 0.10. Tukey's honestly significant difference test revealed a significant difference between the simultaneous and sequential trials in the switch condition (*p* = 0.017), but not in the repeat condition (*p* = 0.999).

The most important finding of Experiment 2 was the different pattern of data between the repeat and switch conditions. In the repeat condition, accuracy was comparable regardless of presentation type. This suggests that, when the appropriate attentional control setting could be used, likely triggered by the target display in the preceding trial (Theeuwes, Kramer, & Belopolsky, 2004), participants could perceive two letters at a time. In contrast, in the switch condition, accuracy was lower in the simultaneous than the sequential trials, indicating that when an appropriate attentional control setting could not be deployed on a trial-by-trial basis performance suffered when the previous trial was a sequential one. Specifically, whereas switching from a simultaneous trial to a sequential one had little impact on performance, switching from a sequential one to a simultaneous one impaired performance. As the target display consisted of two targets in the simultaneous condition, the zoom of attention should be relatively broad as it is influenced by the size of the task relevant region (LaBerge, 1983; LaBerge, Brown, Carter, Bash, & Hartley, 1991). The present results are thus consistent with the notion that an important factor contributing to the effect of selection history was likely to be the extent of attentional zoom, which in turn affected the allocation of attentional resources to the target(s).

To confirm that the processing efficiency of the targets differed between Experiment 2 and Experiment 1B, we performed an independent *t* test on the target exposure duration data from the two experiments. The result showed shorter duration in Experiment 2 than in Experiment 1B, *t*(70) = 3.49, *p* < .001, Cohen's *d* = 0.42, indicating that the alphabet letters in Experiment 2 were indeed processed more efficiently than the geometric shapes in Experiment 1B. Given this result, it seems reasonable to conclude that the pattern of data found in Experiment 2 was the result of the joint effects of efficient target processing and selection history.^3^

## Experiment 3

In Experiment 3, we further explored the role of processing efficiency while controlling for stimulus features. In addition to upright letters, participants performed a shape task involving rotated letters. The two sets of letters have the same physical features, yet observers are likely to be less familiar with the rotated one. If processing efficiency plays a role in the unit of access for shape perception, the pattern of data between the upright and rotated conditions should differ when the letters were presented simultaneously relative to when they were presented sequentially.

###  

#### Method

The experiment used a 2 × 2 mixed design, with Task (upright vs. rotated) as a between-subjects variable and Presentation (simultaneous vs. sequential) as a within-subjects variable. The decision to use different groups of participants for the upright and rotated conditions was to prevent any carryover effects that might affect the results. For the upright group, the method was the same as that of the upright block in Experiment 1A except for the following differences. The target set consisted of 14 letters written in bold, 54-point Consolas font. We used a larger set of stimuli to see whether the unit of access for upright letters would continue to be more than one. The letters were A, B, C, D, E, F, G, K, L, R, T, U, V, and Y in the upright condition. The mask was the same as the one used in Experiment 2. For the rotated group, changes were made to the stimuli. For each of the 14 upright letters used in the upright block, two rotated versions were created, one with a 90° rotation to the left and the other with a 90° rotation to the right. The target set for each participant in the rotated group consisted of seven letters randomly selected from those having a left rotation and seven having a right rotation. Thus, every participant saw 14 different letters across trials. Although the stimuli for those in the upright group all consisted of upright letters, the stimuli for those in the rotated group consisted of seven letters rotated to the left and seven to the right. All of the other aspects of the experiment were the same for the two groups. To keep the number of participants the same as in Experiment 2, we recruited 88 new participants, with half of them randomly allocated to the upright condition and the other half to the rotated condition.

#### Results and discussion

**Figure 4.**
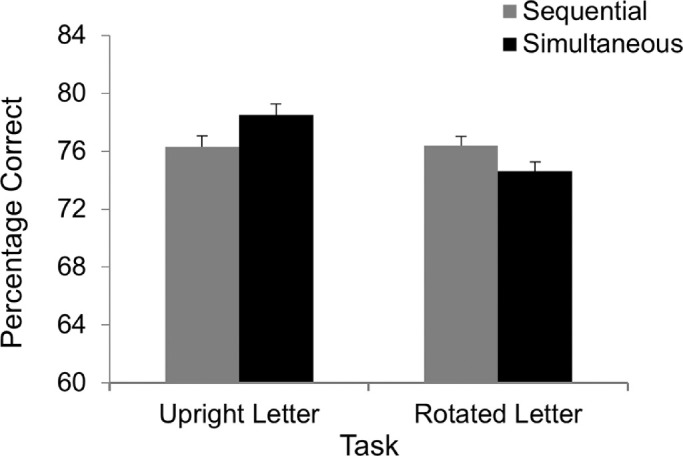
Results from Experiment 3.

The mean target exposure duration was 54.7 ms (range, 43.8–150.9; *SD*, 17.3 ms) for the upright group and 56.5 ms (range, 43.1–101.6; *SD*, 10.0 ms) for the rotated group. Figure 4 shows the accuracy data. A 2 × 2 mixed ANOVA found a main effect of Task, *F*(1, 86) = 9.20, *MS_e_* = 17, *p* = 0.003, η*_p_*^2^ = 0.10, with higher accuracy for upright letters (77.4% correct) than for rotated letters (75.5% correct). There was also a significant interaction between Task and Presentation, *F*(1, 86) = 4.09, *MS_e_* = 42, *p* = 0.046, η*_p_*^2^ = 0.05. Although accuracy was numerically lower in the simultaneous trials (74.6% correct) than in the sequential trials (76.4% correct) for the rotated group, the pattern was reversed for the upright group (78.5% and 76.3% for the simultaneous and sequential trials, respectively). The difference between the two types of trials did not reach significance in either group. The main effect of Presentation was negligible (*F* < 1, not significant).

Experiment 3 found a significant Group by Presentation interaction, which indicates that the effect of Presentation differed between the upright and rotated groups. For the upright group, there was no indication that performance was negatively affected by presenting two letters simultaneously. In fact, accuracy was numerically higher in the simultaneous than sequential trials, suggesting that the unit of access was greater than one, a finding consistent with the result of Experiment 2.

The participants in the rotated group showed a different pattern of data. Interestingly, although accuracy was higher in the sequential trials than in the simultaneous ones, the difference did not reach statistical significance. It is possible that the rotated letters used in the experiment were still relatively familiar to the participants. It is also possible that processing efficiency is affected more by the confusability of features among a set of stimuli than by participants’ familiarity with the stimuli. Because stimulus features were held constant regardless of whether the letters were upright or rotated, the decrease in processing efficiency was limited when the letters were rotated. Thus, despite an overall increase in task demand when the targets were rotated letters compared with upright letters, as indicated by the main effect of Task, the increase was not large enough to limit the processing of rotated letters to one stimulus at a time. We note that the target exposure durations were comparable between the two types of letters in Experiment 3 (54.7 ms and 56.5 ms for upright and rotated letters, respectively; *p* = 0.536), and both were substantially shorter compared with the duration for geometric shapes in the block condition in Experiment 1A (79.6 ms; *p* = 0.007 for upright letters and *p* = 0.010 for rotated letters). This may explain why performance differed significantly between the simultaneous and sequential trials in Experiment 1A but not in the rotated condition in Experiment 3. However, regardless of the exact cause for the pattern of data found in Experiment 3, when taken as a whole the results of the present experiments are consistent with the notion that processing efficiency plays a role in the number of shapes reaching awareness at a given instant.

## General discussion

The present study investigated the limit of visual awareness at a given instant as a function of processing efficiency and selection history in shape perception. When the targets were geometric shapes and their processing was relatively inefficient, accuracy was higher in the sequential than the simultaneous trials, indicating that only one shape was consciously perceived at a time. However, when the targets were upright letters and their processing was more efficient, no difference in performance was found between the simultaneous and sequential trials, suggesting that the unit of access was more than one for upright letters. The results of Experiment 3 further showed that processing efficiency influenced the capacity for shape processing. These results are consistent with those of Rideaux et al. (2015), who reported a more severe limit when the direction of motion was more difficult to discriminate compared with when it was easier to discriminate.

Our study also suggest that selection history may differentially impact the processing of high- and low-efficiency stimuli. Although selection history had negligible influence on the unit of access for geometric shapes in Experiment 1B, it affected the performance for upright letters in Experiment 2. This asymmetry can be explained in the framework of the attentional control setting, with the two key factors being the extent of attentional zoom and the amount of attentional resources a target receives on a given trial.

When stimuli (e.g., geometric shapes) are relatively difficult to discriminate, their processing is inefficient, as their representations must be precise. To achieve high-precision representations, each stimulus must have sufficient attentional resources. In the sequential condition of the present study, this could be done easily. As the targets were shown one at a time, a serial processing strategy could be used to deploy focal attention to each target. In the simultaneous condition, participants could use the same strategy. However, because the target display was shown very briefly, it was unlikely that there would be sufficient time for both targets to be processed to the degree for their representations to reach awareness. Alternatively, participants could use a broader attentional zoom to process the targets in parallel. Unfortunately, with a larger attentional zoom, each target would receive fewer attentional resources (Eriksen & St. James, 1986), and this in turn would lead to decreased quality of the target representations. Thus, when a task requires high-precision representations, regardless of whether a serial or parallel processing strategy was adopted in the simultaneous condition, performance is impaired in that condition compared with the sequential condition. We found this pattern of data in Experiments 1A and 2B.

A different picture emerges when stimuli (e.g., upright letters) are easier to discriminate and the processing efficiency of the targets is high. As the task can be performed on the basis of relatively coarse presentations of the targets, focal attention is unlikely to be needed to encode a target to the degree of making a correct response, especially when there are no other stimuli in the same display to compete for representation (Desimone & Duncan, 1995). When a blocked design was used in Experiment 3, different attentional control settings could be deployed in the simultaneous and sequential blocks. Relative to the sequential block, participants could use a broader attentional zoom, more attentional resources, and greater effort in the simultaneous block. As a result, performance was comparable between the two types of trials.

In Experiment 2, an optimal attentional control setting could not be set up in advance due to the mixing of the simultaneous and sequential trials within a block. If we assume that participants “expected” the same trial type to continue (Theeuwes et al., 2004), they would use the same attentional control setting as in the preceding trial. In the repeat condition, this would have little negative influence on performance regardless of presentation type. However, in the switch condition, this would negatively affect the simultaneous trials but not the sequential ones. Although the attentional control setting for a simultaneous trial (e.g., a relatively broad attention zoom, greater effort) could meet the needs of a sequential one, the reverse was not true. When participants “expected” a sequential trial but encountered a simultaneous one, they either had to adjust the attentional control setting, which would take time, or had to make do with the existing setting, which would be ill suited for the processing needs of the targets. In both cases, performance would suffer in the simultaneous trials relative to the sequential ones. This pattern of data was found in the switch condition of Experiment 2.

The results of the present study are in line with previous research that has shown a trade-off between the quality of stimulus representation and the number of stimuli that are encoded concurrently (Liu & Becker, 2013; Rideaux & Edwards, 2016; Rideaux, Baker, & Edwards, 2018; Ye, Hu, Li, Ristaniemi, Liu, & Liu, 2017; but see Miller et al., 2014). In different experiments, Rideaux and colleagues used a recall task to investigate the cost associated with parallel consolidation of motion direction, orientation, and color. Participants saw two simultaneously or sequentially presented stimuli, a location cue indicating the target, and a probe that could be manipulated by the mouse. The task was to use the mouse to adjust the probe so it indicated the specific feature value of the target. The results show that the offset between the target and response was more variable in the simultaneous condition compared with the sequential condition, suggesting that the target was encoded with reduced precision in the simultaneous condition. Rideaux et al. concluded that the trade-off between the number of items consolidated in parallel and the precision at which they are encoded is a general principal of consolidation in visual working memory. Although our study was not designed to determine whether participants used a serial or parallel processing mode to encode simultaneously presented targets, it is conceivable that the trade-off between quantity and precision could lead to impaired performance in the simultaneous trials in the present study and in some previous studies (but see Liu & Becker, 2013). However, as selection history was found to modulate performance, both in the present study and in Chen and Chen (2021), this may indicate that the trade-off between quantity and precision does not occur under some situations, especially when participants can deploy optimal attentional control settings for a trial either through prior knowledge or selection history. Of course, it is also possible that the trade-off did occur but the effect was negligible in the present paradigm.

It is unclear to what degree processing efficiency contributed to the results in some previous studies that found different unit of access for different feature dimensions. Huang et al. (2007) observed single access for color but parallel access for location. On the one hand, it makes sense for location to differ from color or other non-spatial object features in instant visual conscious access. A large number of studies have shown that location enjoys a special status in visual perception. For example, whereas attending to an object feature such as orientation, color, or form results in the selection of that object's location even when location is task irrelevant (e.g., Cave & Chen, 2017; Chen, 2005; Chen, 2009; Chen & Wyble, 2015; Golomb, Kupitz, & Thiemann, 2014; Humphries, Chen, & Cave, 2021; Kim & Cave, 1995; Tsal & Lavie, 1993), there is no evidence that attending to an object's location leads to the processing of a non-spatial object feature when the latter is not task relevant (Chen, 2005; Chen, 2009; Chen & Wyble, 2015; Golomb et al., 2014; for a review, see Lamy & Tsal, 2001). Location also holds a central place in many theories of attention (e.g., Broadbent, 1982; Cave, 1999; Posner, Snyder, & Davidson, 1980; Treisman & Gelade, 1980; Wolfe, 2021). On the other hand, as the processing of location is usually more efficient than the processing of color (e.g., Huang, 2010), it is possible that processing efficiency may have contributed to some degree to the observed difference in the unit of access for color and location.

In summary, the present study shows that both the processing efficiency of the targets and the participants’ selection history influence the unit of access in shape perception. This finding fits well with the results from previous research on the perception of orientation, motion, and color that spatial attention, similarity between targets, and selection history can all affect the number of stimuli reaching awareness. Together, they indicate that differences in the unit of access may not reflect fundamental differences between different feature dimensions. They also underscore the flexibility of the visual system, which uses a variety of factors to maximize conscious visual perception at a given moment.

## Footnotes

1 The change from black targets on a white background in Experiments 1A and 1B to white targets on a black background in Experiment 2 was not by design. It was the result of creating the experiment by using a pre-existing experiment as a template.

2 We initially set the sample size to 30, which was the same as in Experiments 1A and 1B. After the experiment was completed, we conducted a power test based on the observed effect size. For 80% power, G*Power 3.1 recommended a sample size of 38. To ensure sufficient power, we increased the sample size to 44.

3 Interestingly, when the results of Experiment 1B and Experiment 2 were examined separately, participants showed that selection history affected performance when the processing efficiency was high (Experiment 2) but not when it was low (Experiment 1B). However, a combined analysis on the accuracy data from the two experiments, with Experiment as a between-subjects factor and Trial Type and Presentation as within-subjects factors, found no significant effects involving Experiment. It is unclear to what extent these results could be attributed to the staircase procedure used in the experiments in order to keep participants’ performance at a level of accuracy between 65% and 75% to avoid the floor and ceiling effects. The staircase procedure likely resulted in some of the effects being absorbed by the target exposure duration. (We thank an anonymous reviewer for pointing this out.) Regardless of the cause for the observed pattern of data, the lack of a significant three-way interaction prevents us from reaching a definitive conclusion that selection history differentially impacts high and low efficiency stimuli. Additional studies are needed to further examine the relationship between processing efficiency and selection history.
